# Hidden in plain sight - Multiple resistant species within a strongyle community

**DOI:** 10.1016/j.vetpar.2018.06.012

**Published:** 2018-07-15

**Authors:** Jennifer McIntyre, Kim Hamer, Alison A. Morrison, David J. Bartley, Neil Sargison, Eileen Devaney, Roz Laing

**Affiliations:** a Institute of Biodiversity, Animal Health and Comparative Medicine, University of Glasgow, Garscube Estate, Glasgow G61 1QH, UK; b Royal (Dick) School of Veterinary Studies, University of Edinburgh, Easter Bush Campus, Midlothian, EH25 9RG, UK; c Disease control, Moredun Research Institute, Pentlands Science Park, Bush Loan, Penicuik, EH26 0PZ, UK

**Keywords:** FECRT, Benzimidazole, Ivermectin, Resistance, *Teladorsagia*, Diversity, *In vitro* assays, Pyrosequencing

## Abstract

Ovine parasitic gastroenteritis is a complex disease routinely treated using anthelmintics. Although many different strongyle species may contribute to parasitic gastroenteritis, not all are equally pathogenic: in temperate regions, the primary pathogen is *Teladorsagia circumcincta*. In this study we investigated benzimidazole and ivermectin resistance on a commercial sheep farm in southeast Scotland. We assessed the impact of species diversity on the diagnosis of resistance using the faecal egg count reduction test and *in vitro* bioassays, and correlated the results with the frequency of benzimidazole resistance-associated genotypes measured in the *T. circumcincta* population by pyrosequencing of the β-tubulin isotype-1 gene.

Faecal egg count reduction test results showed efficacies of 65% for albendazole and 77% for ivermectin, indicating moderate resistance levels on the farm. However, PCR speciation of the same populations pre- and post-treatment revealed that removal of susceptible species had masked the presence of a highly resistant population of *T. circumcincta*. Less than 25% of individuals in the pre-treatment populations were *T. circumcincta,* the remainder consisting of *Cooperia curticei, Chabertia ovina, Oesophagostomum venulosum* and *Trichostrongylus* spp. In contrast, post-treatment with albendazole or ivermectin, the majority (88% and 100% respectively) of the populations consisted of *T. circumcincta*. The egg hatch test for benzimidazole resistance and the larval development test for ivermectin resistance were carried out using eggs obtained from the same populations and the results were broadly consistent with the faecal egg count reduction test. Thirty individual *T. circumcincta* from each sampling time point were assessed for benzimidazole resistance by pyrosequencing, revealing a high frequency and diversity of resistance-associated mutations, including within the population sampled post-ivermectin treatment.

These results highlight the potential diversity of parasite species present on UK farms, and their importance in the diagnosis of anthelmintic resistance. On this particular farm, we demonstrate the presence of a highly dual-resistant population of *T. circumcincta*, which was strongly selected by treatment with either benzimidazoles or ivermectin, while other potentially less pathogenic species were removed.

## Highlights

• PCR speciation highlighted parasite species diversity on a commercial UK sheep farm.
• Species diversity confounded interpretation of faecal egg count data and bioassays.
• These tests detected only moderate resistance to benzimidazoles and ivermectin.
• Post-treatment populations were composed almost entirely of *Teladorsagia circumcincta.*
• Ivermectin strongly selected for a highly dual-resistant and pathogenic species.

## Graphical abstract

**Figure:**
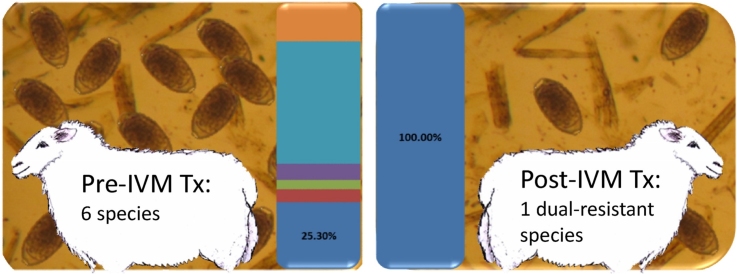

## 1 Introduction

Parasitic gastroenteritis (PGE) is ubiquitous on UK sheep farms and is a significant production limiting disease (Sargison, 2011), resulting in poor weight gain, diarrhoea, dehydration, anaemia and death (Durham and Elliott, 1975). PGE is a complex disease, influenced by many host and management factors and caused by a diverse range of strongyle species (Burgess et al., 2012), of which *Teladorsagia circumcincta* predominates during the UK summer. The clinical diagnosis in live animals is underpinned by faecal egg counts (FECs) to detect and enumerate strongyle eggs.

Broad-spectrum anthelmintics, including benzimidazoles (BZ) and ivermectin (IVM), are used to treat PGE but resistance is widespread (Kaplan and Vidyashankar, 2012). Two recent studies in Wales and Northern Ireland found between 81 and 94% of farms tested had BZ resistance and 50–51% had IVM resistance (McMahon et al., 2013; Hybu Cig Cymru (HCC), 2015). In practice, anthelmintic efficacy is determined using a faecal egg count reduction test (FECRT). This involves faecal sampling a group of animals pre- and post-treatment (Coles et al., 2006), or comparison of a post-treatment group with a control group (Coles et al., 1992). The reduction in strongyle FEC is calculated to determine the anthelmintic efficacy. If the reduction is less than 95%, and the lower 95% confidence interval is less than 90%, anthelmintic resistance is diagnosed (Coles et al., 1992).

The FECRT is a straightforward and useful test, but it has limitations. FECRTs depend on a reduction in strongyle eggs, but these generally consist of a mixed population of strongyle species. Importantly, not all strongyles are considered equally pathogenic in sheep (Crilly and Sargison, 2015), and not all species on a holding are necessarily drug resistant (McKenna, 1997; McMahon et al., 2013; Melville et al., 2016). The FECRT also lacks sensitivity if resistant phenotypes comprise less than 25% of the population (Martin et al., 1989). This phenomenon has been noted in the disparity between BZ resistance genotypes detected by PCR and FECRT results (Kaplan and Vidyashankar, 2012). BZ resistance is associated with non-synonymous single nucleotide polymorphisms (SNPs) within the β-tubulin isotype-1 gene, which appear conserved within and between strongyle species and can be detected by PCR or pyrosequencing (Kwa et al., 1995; Silvestre and Cabaret, 2002; Ghisi et al., 2007; Redman et al., 2015). Molecular tests are highly sensitive and specific, but are only currently available for BZ resistance. While laboratory bioassays for anthelmintic resistance are a possible alternative, they are time consuming and can be technically challenging in mixed species infections.

This study describes a FECRT undertaken on a commercial sheep farm in southeast Scotland to compare BZ and IVM resistance status detected in the field, combined with *in vitro* phenotypic bioassays and pyrosequencing of the β-tubulin isotype-1 gene. The results highlight the importance of parasite species composition in the interpretation of FECRTs and in the design of appropriate and sustainable control strategies.

## 2 Materials and methods

### 2.1 Study Farm

A lowland sheep farm in southeast Scotland was visited in September 2016, with the agreement of the shepherd and previous knowledge of the presence of anthelmintic resistance (Wilson et al., 2008; Sargison et al., 2012). Each year in the autumn, a proportion of the breeding flock was replaced with bought in ewe lambs. Sheep were able to graze rented pastures, which were also leased to neighbouring sheep farms. In addition, cattle occasionally grazed farm pastures, though had not done so in the last two years. Moxidectin is used routinely at lambing time, with most twin-bearing ewes treated and some triplet- or single-bearing ewes also treated. BZs are used to control *Nematodirus battus* in the lambs, with three treatments used in the year of study. Further anthelmintics are used to control summer PGE in the lambs, and rams are dosed periodically with anthelmintics. Table 1 shows the anthelmintics given during the year of study.

**Table 1:** Anthelmintics administered during the year 2016 from lambing until all lambs were sold.

Date of treatment	Anthelmintic	Treatment group
1^st^ April onwards – at lambing	Moxidectin	Lambed ewes
23/05/16	Albendazole	Lambs
14/06/16	Albendazole	Lambs
01/07/16	Ivermectin	Rams
06/07/16	Albendazole	Lambs
20/09/16	Levamisole	Lambs
20/09/16	FECRT – albendazole or ivermectin	Two groups of lambs

### 2.2 Faecal egg count reduction test

Thirty-five, five-and-a-half month old lambs were set aside by the farmer and from these, animals were selected randomly for use in the FECRT. Faeces were collected per rectum for FECs pre-treatment, with additional material gathered for further analysis, if voided immediately following treatment. Ten lambs were allocated to the albendazole (BZ) treatment group (5 mg/kg body weight (BW), Albex™ 2.5% w/v SC oral suspension (Chanelle UK)) and twelve lambs to the IVM treatment group (0.2 mg/kg BW, Noromectin® 0.08% w/v Drench Oral Solution (Norbrook)). Lambs were identified to their group but not individually. All were weighed individually, using the shepherd’s weigh scales (EID weigh crate, Shearwell), and dosed *per os* according to their individual weights using a syringe. Repeat faecal samples were collected on days 7 and 14 post-treatment and faeces transferred anaerobically to the laboratory.

A cuvette method, sensitive to 1 egg per gram (epg) (Christie and Jackson, 1982) was used for the FECs. Briefly, faeces were weighed and water was added in a ratio of 10 ml to 1 *g*, following which 10 ml was strained through a coarse sieve (1 mm aperture). After centrifugation at 234 ×g for 2 min, the supernatant was aspirated and the egg pellet re-suspended in saturated sodium chloride solution (specific gravity 1.2) with a further centrifugation step (as above). The meniscus was isolated using artery forceps and tipped into a cuvette, which was filled with saturated NaCl solution for counting. Strongyle egg counts were analysed using Minitab (Minitab® 17.1.0, Microsoft). Group arithmetic means were calculated, with 95% confidence intervals (CIs). Differences between medians of sample groups were assessed for significance by a Mann-Whitney test. The FECR percentage and Bayesian CIs were calculated using R Shiny ‘eggCounts’ web interface (Wang and Paul, 2017; Wang et al., 2017).

### 2.3 Bioassays

Strongyle eggs were harvested for immediate use in bioassays (within three hours of faecal collection) as described in 2.2, but with the following modifications; emulsified faeces were strained through a series of sieves, (215 μm, 125 μm and 63 μm), with eggs collected from a 38 μm sieve. Centrifugation steps were performed as described above, and the meniscus was poured back onto a small, 38 μm sieve. Eggs were washed with tap water to remove salt and collected. A 200 μl aliquot of egg suspension was counted, using the cuvette method, to determine the total number of eggs/ml. The egg concentration was adjusted to 1 egg/μl for the egg hatch test (EHT) and 2 eggs/μl for the larval development test (LDT).

#### 2.3.1 Egg hatch test

An EHT was set up to measure BZ efficacy, essentially as described previously (Coles et al., 2006). Stock solutions of TBZ were prepared by diluting 1 mg/ml of TBZ/DMSO solution into DMSO and were stored in opaque tubes. Briefly, a 24 well plate was set up with a range of final TBZ concentrations as follows: 0.05 μg/ml, 0.1 μg/ml, 0.2 μg/ml, 0.3 μg/ml, 0.4 μg/ml, 0.5 μg/ml, 1 μg/ml and 0.5% DMSO control (Sigma Aldrich). To each well was added: 10 μl of TBZ/DMSO solution, 1890 μl deionised water (Acros Organics) and 100 μl of egg suspension, giving approximately 100 eggs/well.

Four EHTs were performed using pre-treatment samples for each of the BZ and IVM treated lamb groups. For each of the eight tests, all concentrations were repeated in triplicate, when sufficient eggs were available. Plates were incubated in a humid environment at 25 °C for 48 h and the number of unhatched eggs and first stage larvae (L1) recorded at 48 h.

On day 14 post-treatment a reduced EHT was performed for the BZ treated population as follows: all TBZ concentrations were incorporated but a reduced quantity of eggs restricted replicate numbers. Two replicates were carried out for each of 0.05 μg/ml, 0.1 μg/ml and 0.2 μg/ml. The rest were single wells. Due to a paucity of eggs post-IVM treatment of lambs, it was not possible to perform a full EHT and only two replicates of the ‘definitive dose’ wells (0.1 μg/ml) were included.

The ED_50_, the effective dose at which 50% of larvae fail to hatch, was calculated using a binomial (probit) general linear model (GLM) in R Studio (version 1.1.383 – © 2009–2017 RStudio, Inc; R version 3.4.3 © 2017). All data were first corrected for the percentage hatch in the DMSO control wells and the TBZ concentrations were log_10_ transformed. If the ED_50_ was greater than 0.1 μg/ml TBZ, then the sample population was considered resistant to BZ (Coles et al., 1992; von Samson-Himmelstjerna et al., 2009).

#### 2.3.2 Larval development test

The LDT was performed on strongyle populations obtained pre- and post-IVM treatment (day 0 and day 14) and pre-BZ treatment. Insufficient eggs were recovered post-BZ treatment to allow a LDT to be carried out. A method adapted from Varady et al (1996) was used to ascertain the ability of larvae to develop in the following concentrations of IVM: 0.57 nM, 1.14 nM, 2.29 nM, 4.54 nM, 9.14 nM, 18.29 nM and 36.58 nM, water only (control), and 2% DMSO (control). Stock solutions of IVM (Catalogue number I8898, Sigma Aldrich) were prepared by diluting a 0.001 M IVM/DMSO solution into DMSO and were stored in opaque tubes. Briefly, a 48 well plate was set up, each well containing 6 μl of IVM/DMSO solution, 239 μl deionised water and 5 μl of a highly concentrated, reconstituted, lyophilised *E. coli* OP50 as a food source for the developing larvae (Heim et al., 2015). 50 μl of strongyle eggs were added (concentration of 2 eggs/μl), giving approximately 100 eggs/well. No antimicrobials were used. Wells surrounding those used for the assay were filled with deionised water and the plate sealed with an adhesive plastic seal (Sigma Aldrich). The plate lid was replaced and the assay incubated for 7 days at 25 °C in a humid environment. Eggs, L1/L2 and L3 larvae were counted. Following correction for development in the control wells, an ED_50_ for each sample was calculated using a general linear model (GLM) constructed in R Studio as above.

### 2.4 Molecular biology

#### 2.4.1 Lysates

Lysates were made from individual strongyle eggs, L1 or infective larvae (L3) in 96 well plates from all sampling time points. On day 0 and day 7 post-treatment, L3 from coproculture were used. On day 14 post-treatment, eggs and L1 from EHTs were used. Briefly, a master mix per 100 wells was prepared as follows: 1000 μl DirectPCR Lysis Reagent (Cell; Viagen Biotech), 50 μl 1 M DTT (Thermofisher Scientific) and 10 μl Proteinase K (Fungal; Invitrogen) at 100 mg/ml in DEPC treated water (Ambion). 10 μl was dispensed per well and a single egg or larva added to each in ≤ 1 μl of water/PCR Lysis Reagent. After incubation at 60 °C for 2 h, followed by 85 °C for 45 min to denature the proteinase K, lysates were aliquoted in 1:20 dilutions using DEPC treated water. All lysates were stored at −80 °C.

#### 2.4.2 Speciation of strongyles by PCR

Individual eggs, L1 or L3, harvested as described in 2.4.1, were identified by PCR to species level using the ITS2 region (Wimmer et al., 2004; Redman et al., 2008; Burgess et al., 2012; Bisset et al., 2014). Strongyle numbers speciated were as follows: for the BZ FECRT on day 0, 96 L3, on day 7, 62 L3 and on day 14, 82 eggs or L1. For the IVM FECRT on day 0, 83 L3 were speciated, on day 7, 83 L3 and on day 14, 42 eggs or L1. Two PCR methods were employed. Briefly, single species PCR was performed using GoTaq Flexi polymerase (Promega) according to the manufacturer’s instructions. The total volume of each reaction was 12.5 μl of which 1 μl was 1:20 DNA lysate. Primers designed to amplify the ITS2 region and specific to the species of interest were used. Table 2 shows primer sequences with their original reference, expected amplicon size, and T_A_ °C. All PCRs were carried out using the following protocol: denaturation at 94 °C for 2 min, then 35 cycles each of 94 °C 30 s, T_A_ °C 30 s and 72 °C 30 s, with a final extension step of 72 °C 10 min.

**Table 2:** Primer sequences used in this study.

Species	Primer name	Sequence	T_A_ (°C)	Label	Product size (bp)	Reference
*Teladorsagia circumcincta*	Tc_ITS2	F: ATACCGCATGGTGTGTACGG	52		421	Burgess 2012
		R: CAGGAACGTTACGACGGTAAT				Burgess 2012
*Trichostrongylus vitrinus*	Tv_ITS2	F: AGGAACATTAATGTCGTTACA	52		100	Wimmer 2004
		R: CTGTTTGTCGAATGGTTATTA				Wimmer 2004
*Haemonchus contortus*	Hc_ITS2	F: GTTACAATTTCATAACATCACGT	50		321	Redman 2008
		R: TTTACAGTTTGCAGAACTTA				Redman 2008
Generic ITS2	ITS2GF	F: CACGAATTGCAGACGCTTAG	54		370-398	Bisset 2014
Generic ITS2	ITS2GR	R: GCTAAATGATATGCTTAAGTTCAGC	54			Bisset 2014
*Trichostrongylus axei*	TraxFd2	F: GATGTTAATGTTGAACGACATTAATATC	52		186	Bisset 2014
*Chabertia ovina*	ChovFd2	F: CAGCGACTAAGAATGCTTTGG	54		115/117	Bisset 2014
*Cooperia curticei*	CocuFd3	F: TAATGGCATTTGTCTACATTGGTTC	53		252	Bisset 2014
*Oesophagostomum venulosum*	OeveRv1	R: CGACTACGGTTGTCTCATTTCA	54		323-329	Bisset 2014
*Teladorsagia circumcincta*	Btub_SK_200_FOR	F: ACCTTACAATGCCACTCTTTCTG	55	Biotin	97	Skuce 2010
*Teladorsagia circumcincta*	Btub_SK_200_REV	R: GCGGAAGCAGATATCGTACAG	55			Skuce 2010
*Teladorsagia circumcincta*	Btub_SK_200_SEQ	SEQ: RGAGCYTCATTATCGATR				Skuce 2010
*Teladorsagia circumcincta*	Btub_SK_167_FOR	GCATTCTTTGGGAGGAGGTA	55		122	Skuce 2010
*Teladorsagia circumcincta*	Btub_SK_167_REV	TGCACCTCGAGAACCTGTACATA	55	Biotin		Skuce 2010
*Teladorsagia circumcincta*	Btub_SK_167_SEQ	CGGATAGAATCATGGCT				Skuce 2010

Multiplex PCR was also performed, adapted from a protocol optimised by Bisset et al. (2014). Briefly, each 12.5 μl reaction contained: 2.5 μl 5X GoTaq Green buffer, 1.25 μl MgCl_2_ 25 mM, 0.0625 μl GoTaq Flexi polymerase 5 U/μl (Promega), 0.25 μl dNTPs 10 mM each (NEB Inc.), 0.15 μl Generic Forward primer 10 μM, 0.15 μl Generic Reverse primer 10 μM, 0.25 μl *Trichostrongylus axei* Forward primer 10 μM, 0.35 μl *Cooperia curticei* Forward 3 primer 10 μM, 0.1 μl *Oesophagostomum venulosum* Reverse 1 primer 10 μM, 0.15 μl *Chabertia ovina* Forward 2 primer 10 μM, (Eurofins Genomics) and 6.2875 μl DEPC treated water (Ambion). To this 1 μl of diluted DNA lysate was added. Touchdown PCR conditions were: denaturation at 94 °C for 2 min, then 12 cycles each of 94 °C 15 s, 60 °C, decreasing by 0.5 °C each cycle, to 54.5 °C for 15 s and lastly 72 °C 30 s. Next, 25 cycles each of 94 °C 15 s, 54 °C 15 s, 72 °C 30 s, with a final extension step of 72 °C for 7 min. Products were visualised with SafeView (NBS Biologicals) on 2% agarose gels for single species PCR and 2.5% agarose gels for multiplexed PCR.

Any unidentified strongyles (*i.e.* positive on the multiplex PCR as a strongylid nematode) were amplified with Phusion High-Fidelity Polymerase (NEB Inc.) using the generic ITS2 primer set (Bisset et al., 2014) and sequenced by Eurofins Genomics. A BLAST search using NCBI was performed for identification. Speciation results were analysed in Microsoft Excel and proportions of each species, determined by PCR, were used to estimate individual *T. circumcincta* egg counts pre- and post-treatment. A 2-sample proportion test was performed in Minitab to compare speciation results pre- and post-treatment.

#### 2.4.3 Pyrosequencing

Using a method adapted from Skuce et al. (2010), BZ resistance associated SNPs were pyrosequenced in 30 *T. circumcincta* individuals from each sampling time point (eggs, L1 or L3, as described in 2.4.1), providing a total of 180 individuals for analysis. As for the speciation PCRs, on days 0 and 7 of each FECRT, 30 L3 were selected, but on day 14 post-treatment for each FECRT, a combination of eggs and L1 were used, totalling 30 individuals. Two regions of the β-tubulin isotype-1 gene (one region including codons 200 and 198, and the other including codon 167) were amplified using the Pyromark PCR kit (Qiagen) according to the manufacturer’s instructions. Briefly, a 25 μl reaction was set up using 12.5 μl 2X Pyromark Master Mix, 0.5 μl of each primer, 10.5 μl DEPC treated water (Ambion) and 1 μl of diluted DNA lysate. PCR conditions were: denaturation at 95 °C for 15 min, then 45 cycles of 94 °C for 30 s, 55 °C for 30 s and 72 °C for 30 s, with a final extension step of 72 °C for 10 min. Pyrosequencing was performed on a Biotage Q96 ID instrument (Qiagen) according to the manufacturer’s protocol with pyrograms evaluated manually. The sequences analysed were; 200/198 ‘CAGWAYGTYDMRTCGG’ for codons F200Y and E198 A/L, and ‘TCATWCTC’ for codon F167Y. Primers used are shown in Table 2. A 2-sample proportion test was performed in Minitab to compare pyrosequencing results pre- and post-treatments.

## 3 Results

### 3.1 FECRT indicates resistance to anthelmintics tested

The FECRTs for both BZ and IVM indicated the presence of anthelmintic resistance (Table 3). The BZ treated lambs showed a reduction of 65% (95% Highest Posterior Density (HPD) Interval: 14.5, 86.2) in their strongyle faecal egg output at day 14 and the IVM group, a 77% (95% HPD Interval: 45.5, 91.6) reduction in faecal egg output.

**Table 3:** Benzimidazole and ivermectin faecal egg count reduction tests. Arithmetic mean strongyle egg counts and proportional *Teladorsagia circumcincta* egg counts (adjusted by species percentage as determined by PCR) are reported, with strongyle faecal egg count reduction percentages for each anthelmintic. The benzimidazole faecal egg count reduction percentage has been calculated for both *T. circumcincta* and *Cooperia curticei* but only for *T. circumcincta* post ivermectin treatment as all other species egg counts were zero on day 14 post-ivermectin.

Sample ID	N^a^	Mean Strongyle epg^b^	SD Strongyle epg	Mann-Whitney test Strongyle epg^c^		Proportional *T. circumcincta* Mean epg^d^	SD *T. circumcincta* epg	Mann-Whitney test *T. circumcincta* epg^b^		Strongyle FECR % (Bayesian 95 % HPD^e^ Interval)^f^	*Teladorsagia circumcincta* FECR % (Bayesian 95 % HPD Interval) ^d^	*Cooperia curticei* FECR % (Bayesian 95 % HPD Interval)^d^
				D7	D14			D7	D14			
BZ D0	10	209	182	**p = 0.005**	**p = 0.005**	41.3	36	p = 0.056	p = 0.212	–	–	–
BZ D7	8	47.1	75.4	–	p=0.534	21.3	34	–	p = 0.625	–	–	–
BZ D14	10	77	156	–	–	67	137	–	–	65.3 (14.5, 86.2)	4.99 (0.0, 55.8)	91.8 (82.4, 96.1)
IVM D0	12	202	186	**p = 0.003**	**p = 0.002**	51	47	p = 0.976	p = 0.583	–	–	–
IVM D7	11	53.8	53.6	–	p = 0.666	53.8	53.6	–	p = 0.666	–	–	–
IVM D14	12	48.4	57.9	–	–	48.4	57.9	–	–	77.2 (45.5, 91.6)	9.17 (0.0, 53.2)	–

^a^Number of lambs sampled. Lambs were brought in from the field at each sampling time point by the farmer. On day 7 not all lambs were present or contained sufficient material per rectum for an adequate sample to be obtained.

^b^Egg per gram.

^c^Comparing egg count medians between sampling dates; p value reported. Ran ‘Difference = η (D0) – η (D14)’, 95% confidence intervals, hypothesis of ‘medians not equal’ using Minitab. Numbers in bold indicate significant differences between sample medians.

^d^*T. circumcincta* epg calculated for each individual sample using the strongyle species proportions obtained from PCR of the ITS2 region of L3 (D0, D7) and eggs/L1 (D14).

^e^Highest posterior density interval.

^f^FECR percentage calculated using R shiny ‘eggCounts’, reported HPD.Low, mode and HPD.High as most accurate estimates. Bayesian CI (HPD.Low and HPD.High) are in brackets.

### 3.2 Speciation allows interpretation of the FEC by species of interest

**Fig. 1:**
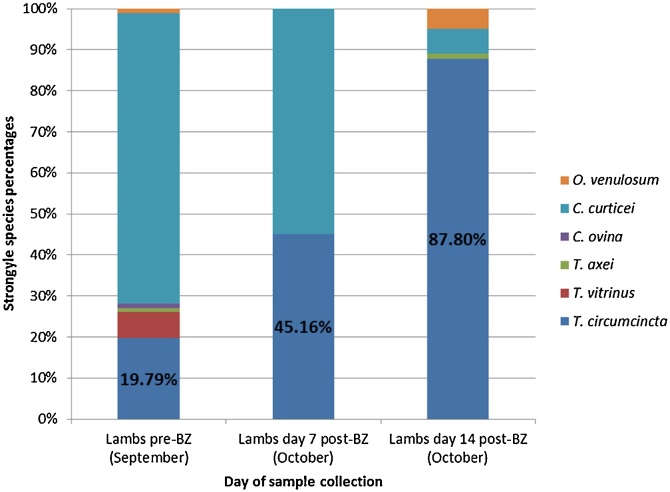
Benzimidazole faecal egg count reduction test: strongyle species percentages. Numbers speciated by PCR: 96 Pre-BZ, 62 day 7 Post-BZ and 82 day 14 Post-BZ. Species identified: *Oesophagostomum venulosum, Cooperia curticei, Chabertia ovina, Trichostrongylus axei, Trichostrongylus vitrinus, Teladorsagia circumcincta.*

**Fig. 2:**
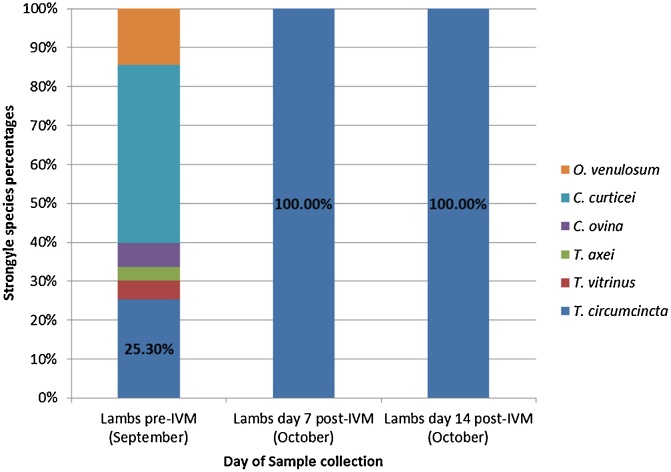
Ivermectin faecal egg count reduction test: strongyle species percentages. Numbers speciated by PCR: 83 Pre-IVM, 83 day 7 Post-IVM and 42 day 14 Post-IVM. Species identified: *Oesophagostomum venulosum, Cooperia curticei, Chabertia ovina, Trichostrongylus axei, Trichostrongylus vitrinus, Teladorsagia circumcincta.*

Speciation by PCR of ≤ 96 strongyle eggs or larvae was performed both pre- and post-treatment. Samples analysed from both groups of lambs contained between 20 to 25% *T. circumcincta* pre-treatment with the remaining 75 to 80% composed of a diverse mix of other strongyle species (Fig. 1, Fig. 2). In faecal samples from the BZ treated lambs, both *T. circumcincta* and *C. curticei* were present at 7 days post-treatment, in roughly equal proportions. By day 14 post-treatment, the strongyle population post-BZ was composed of 88% *T. circumcincta* with the remaining 12% comprising a mixture of *C. curticei, O. venulosum and T. axei* (Fig. 1). In contrast, post-IVM treatment only *T. circumcincta* was present at day 7 and at day 14 (Fig. 2).

### 3.3 Adjustment of the mean FEC by species improves interpretation of the FECRT

**Fig. 3:**
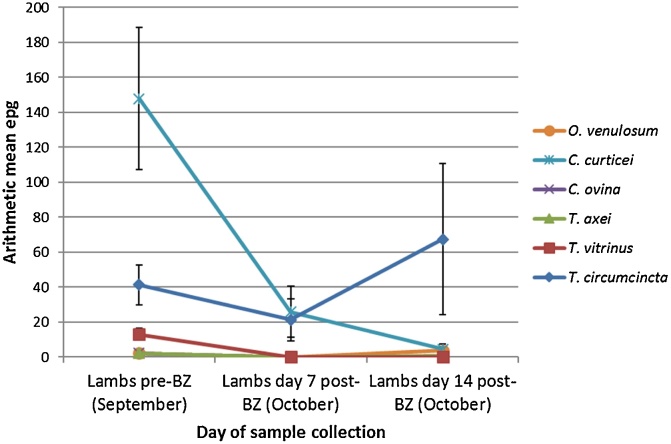
Benzimidazole faecal egg count reduction test: arithmetic mean strongyle egg per gram adjusted by species percentage. Mean strongyle faecal egg counts were adjusted proportionally based on the strongyle species percentage data as determined by PCR. SEM bars are displayed. Species identified: *Oesophagostomum venulosum, Cooperia curticei, Chabertia ovina, Trichostrongylus axei, Trichostrongylus vitrinus, Teladorsagia circumcincta.*

**Fig. 4:**
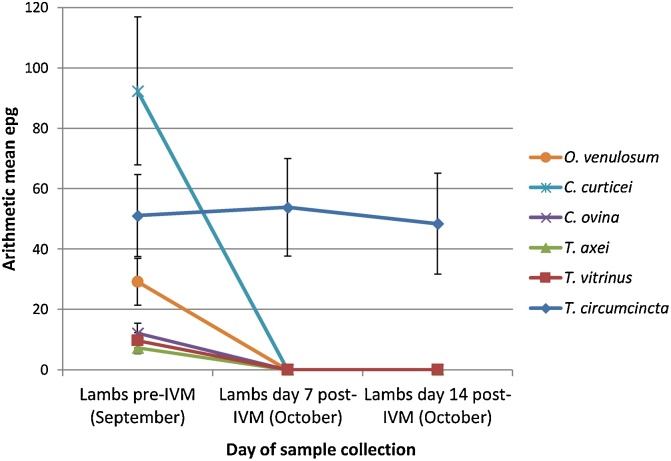
Ivermectin faecal egg count reduction test: arithmetic mean strongyle egg per gram adjusted by species percentage. Mean strongyle faecal egg counts were adjusted proportionally based on the strongyle species percentage data as determined by PCR. SEM bars are displayed. Species identified: *Oesophagostomum venulosum, Cooperia curticei, Chabertia ovina, Trichostrongylus axei, Trichostrongylus vitrinus, Teladorsagia circumcincta.*

An adjustment of the mean FEC by PCR speciation data shows a decrease of approximately 50% in the *T. circumcincta* egg output, and an 82% reduction in *C. curticei* egg output on day 7 following BZ treatment (Fig. 3). No other species were found at day 7 post-BZ treatment. On day 14 post-BZ treatment, *T. circumcincta* egg output had risen considerably relative to day 0, being estimated at 67 epg by speciation. This was not, however, statistically significant. *C. curticei* epg had fallen to 5 epg from an initial 148 epg on day 0. Overall, there was a significant reduction in egg output from strongyle species other than *T. circumcincta* (p = 0.00). Following IVM treatment on the same farm there was no change in egg output for *T. circumcincta*, whilst the egg output of all other species was reduced to zero (Fig. 4).

### 3.4 Phenotyping bioassays (TBZ EHT, IVM LDT)

An EHT (assessing BZ resistance) and a LDT (assessing IVM resistance) were performed on days 0 and 14 to compare FECRT results with *in vitro* phenotype.

#### 3.4.1 Egg hatch test

**Fig. 5:**
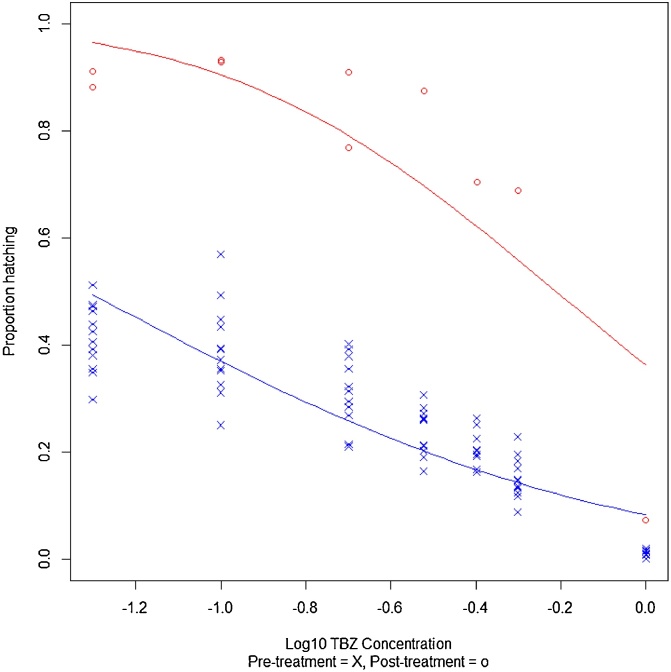
Egg hatch test: pre- and post-benzimidazole faecal egg count reduction test. The corrected EHT data is shown, with log_10_(thiabendazole concentration) plotted against the proportion hatching. The data was modelled in R using a binomial GLM (probit), and the regression line is plotted here.

Pre-BZ treatment 79% of eggs hatched in control wells compared to 91% post-BZ treatment (Supplementary Table 1). Just 39% of eggs from the pre-BZ treatment sample hatched in the TBZ EHT at the definitive dose of 0.1 μg/ml (Coles et al., 2006), while post-BZ treatment, 93% hatched (Table 4), giving a resistance ratio of 2.4 (% hatch post-treatment/% hatch pre-treatment). Calculating the ED_50_ also revealed a difference pre- and post-BZ treatment, with 50% hatching at a higher concentration of TBZ/DMSO post-treatment (0.615 μg/ml) compared with pre-treatment (0.048 μg/ml) (Table 4, Fig. 5), giving a resistance ratio of 12.8 (ED_50_ post-treatment/ED_50_ pre-treatment).

**Table 4:** Egg hatch test results: Effective doses (ED_50_) of thiabendazole required to prevent hatching of 50% of eggs are reported for each full assay performed. The average percentage hatch in definitive dose wells (0.1 μg/ml) is given for each assay.

Assay	EHT ED_50_ (μg/ml)^a^	Percentage hatch in 0.1 μg/ml^b^
Pre-BZ FECRT (four plates combined analysis)	0.048 (0.045, 0.052)	39.1% (8.58)
Post-BZ FECRT	0.615 (0.538, 0.730)	93.1% (0.23)
Pre-IVM FECRT (four plates combined analysis)	0.043 (0.040, 0.046)	32.6% (6.06)
Post-IVM FECRT	–	82.4%^c^ (3.43)

^a^Raw data was adjusted for response in 0.5% DMSO control wells. 95% confidence intervals reported in brackets.

^b^Adjusted for response in 0.5% DMSO control wells. Standard deviation reported in brackets.

^c^No DMSO well was included post-IVM treatment and only two replicate wells for 0.1 μg/ml were included. This therefore is the uncorrected percentage hatch. The uncorrected percentage hatch in the 0.1 μg/ml wells pre-IVM treatment was 27.52% (5.0 standard deviation).

While a full EHT was not carried out for pre- and post-IVM samples, 33% of eggs in the pre-IVM treatment population hatched at 0.1 μg/ml TBZ and an ED_50_ of 0.043 μg/ml was calculated, comparable to the pre-BZ FECRT EHTs (Table 4 and Supplementary Fig. 1). Post-IVM treatment only two replicates of 0.1 μg/ml TBZ were set up, in which 82% eggs hatched, suggesting a resistance ratio of 3.0 at the definitive dose (raw data used, Table 4). This initially surprising result was illuminated by the inclusion of speciation and pyrosequencing data (section 3.5), which revealed that this apparent selection for BZ resistance by IVM instead reflected the presence of a highly dual-resistant *T. circumcincta* population.

#### 3.4.2 Larval development test

**Fig. 6:**
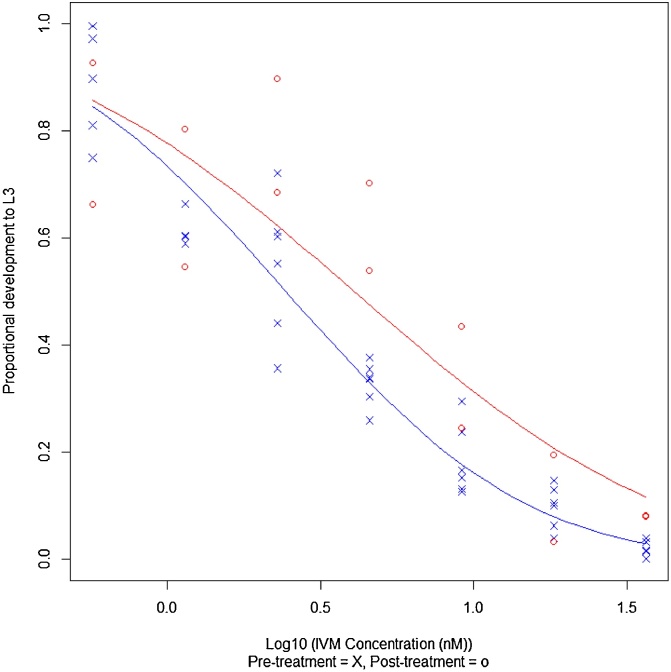
Larval development test: pre- and post-ivermectin faecal egg count reduction test. The corrected LDT data is shown, with log_10_(ivermectin concentration) plotted against the proportion developing to L3. The data was modelled in R using a binomial GLM (probit), and the regression line is plotted here.

Average egg hatch in the 2% DMSO control wells of the LDT was 97%. Pre-treatment 67% of hatched larvae developed to L3 on average (range 53–75%), compared to 86% post-IVM treatment (Supplementary Table 2). No definitive dose has been established for the IVM LDT used in our study, but using a GLM, the ED_50_ can be determined. Pre-IVM treatment this was 2.43 nM (95% CI: 2.14, 2.81) and post-IVM treatment this rose to 4.07 nM (95% CI: 2.87, 6.36) (Table 5 and Fig. 6), giving a resistance ratio of 1.7. Pre-BZ treatment an ED_50_ of 2.27 nM was calculated, comparable to that of the pre-IVM group (Table 5 and Supplementary Fig. 2).

**Table 5:** Ivermectin larval development tests. Effective doses (ED_50_) of ivermectin required to inhibit 50% development of eggs to L3 are reported for each assay performed.

Assay	IVM LDT ED_50_ (nM)^a^
Pre-BZ FECRT (two plates combined analysis)	2.27 (2.05, 2.56)
Pre-IVM FECRT (two plates combined analysis)	2.43 (2.14, 2.81)
Post-IVM FECRT	4.07 (2.87, 6.36)

a Raw data was adjusted for response in 2% DMSO control wells. 95% confidence intervals reported in brackets.

### 3.5 Pyrosequencing of the T. circumcincta β-tubulin isotype-1 gene reveals a diverse range of resistance alleles

**Fig. 7:**
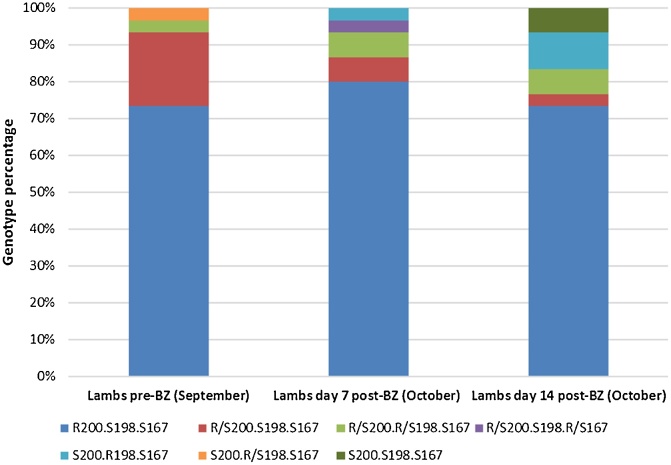
Benzimidazole faecal egg count reduction test: pyrosequenced genotypes of β-tubulin isotype-1. Thirty *T. circumcincta* individuals were sequenced at each sampling time point at codons 167, 198 and 200 of the β-tubulin isotype-1 gene.

**Fig. 8:**
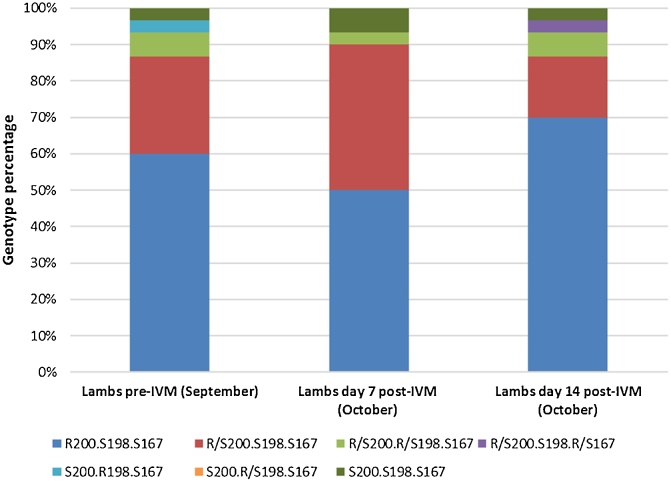
Ivermectin faecal egg count reduction test: pyrosequenced genotypes of β-tubulin isotype-1. Thirty *T. circumcincta* individuals were sequenced at each sampling time point at codons 167, 198 and 200 of the β-tubulin isotype-1 gene.

Previous studies have identified mutations in three codons within the β-tubulin isotype-1 gene that are correlated with BZ resistance (F200Y, E198 A/L, F167Y) (Kwa et al., 1995; Barrère et al., 2012; Kotze et al., 2012). In total, 180 individual *T. circumcincta* from all FECRT sample populations (30 individuals from each sampling time-point) were pyrosequenced at all three codons (Fig. 7, Fig. 8). Of these, 168 were either homozygous or heterozygous for the resistance mutation at codon 200. In total, seven different allelic sequences were identified in the 123 individuals homozygous for this mutation. Individuals that were heterozygous at one or more resistance-associated codons were also detected at all sampling time points of both the BZ and IVM FECRT. Those with homozygous resistance mutations at codon 198 (P200 F, P198 L, P167 F) were detected on day 0 of the IVM FECRT and on days 7 and 14 of the BZ FECRT. All larvae with resistance-associated polymorphisms at codon 198 encoded leucine rather than alanine. No individuals with homozygous resistance mutations at codon 167 were identified, nor were any individuals identified with mutations at other codons if homozygous resistance mutations were present at either codon 198 or 200. Individuals with homozygous susceptible genotypes were identified (genotype P200 F, P198E and P167 F) on days 0, 7 and 14 of the IVM FECRT and on day 14 of the BZ FECRT (Fig. 7, Fig. 8). For both the BZ and IVM treatment groups no significant differences in genotype proportions were noted between samples before and after treatment (Fig. 7, Fig. 8).

## 4 Discussion

The results presented in this paper provide evidence of a dual-resistant *T. circumcincta* population on a commercial farm and highlight the importance of speciation for the valid interpretation of FECRTs. Group mean FECs reduced following both BZ and IVM treatments, by 65% and 77% respectively, suggesting that both IVM and BZ retained some activity on the farm in question. However, extrapolation of the strongyle FEC by individual species revealed that, despite a significant reduction in total FEC post-treatment (p < 0.01), there was no significant change in the egg output of *T. circumcincta,* the primary pathogen causing PGE, following either treatment. The clinical interpretation of this full FECRT is therefore very different to a statistical one based on FECs alone. While high BZ and IVM resistance would be significant on any sheep farm, in this flock, there is also levamisole and moxidectin resistance diagnosed by FECRT (data not shown), leaving the shepherd with very few options for chemotherapeutic control of the nematode population. In this instance an anthelmintic, though < 95% effective, might still be employed as part of a flock health plan to control PGE.

PCR speciation of L3 larvae revealed a diverse mix of strongyle species pre-treatment, a situation not uncommon in the UK (Burgess et al., 2012; Redman et al., 2015). Species diversity may be supported on this farm for several reasons. Firstly, it is an openly managed flock, which may allow influx of new species over time. Previous studies in France have found links between increased species diversity and the number of farms originally contributing to a subsequently closed herd (Silvestre et al., 2000). There are a variety of grazing areas on the farm, including short grass pasture, longer grassy areas containing other weed species and woodland. These may favour micro-niches for parasite species, encouraging diversity (Sinclair et al., 2016). Sheep groups move between pastures and rams rotate between both pastures and sheep groups enabling further mixing of the parasite communities. In addition, cattle have previously grazed on farm pastures, though not in the last two years, a practice that has been shown to increase species diversity on irrigated pasture in the tropics (Giudici et al., 1999). Lastly, the farm is moderate in area, being only about 60 ha, enabling frequent grazing of all pastures, which has been linked in other studies with increased species diversity, due in part to the ability of rarer species with longer pre-patent periods and shorter pasture survival times to be effectively maintained within the flock (Silvestre et al., 2000).

Several species, namely *C. curticei, O. venulosum* and *T. axei,* were also found to be present post-BZ treatment in addition to *T. circumcincta.* This suggests some level of resistance within these populations, consistent with previous studies (Hunt et al., 1992; Moreno et al., 2004; Palcy et al., 2010; Keegan et al., 2017). In contrast to the BZ FECRT, only *T. circumcincta* was detected post-IVM treatment, suggesting that other strongyle species present on the farm are susceptible to IVM, despite apparent resistance to BZ. The pattern of anthelmintic usage on the farm over the year may impact resistance development following treatment with BZ or IVM. BZ anthelmintics were routinely used several times in the spring to control *N. battus,* at a time point when pasture larvae are predominantly present from overwintered survival (Gibson and Everett, 1972), lamb strongyle FECs were minimal and species prevalence diversity was low within the lambs (being principally *T. circumcincta*, data not shown). At this time, and with monthly dosing treatment, BZ resistance may be more efficiently selected within the different species populations due to the smaller pasture population *in refugia* (Martin et al., 1981). In comparison, IVM is used for the most part in late summer, when pasture larval levels are higher and egg counts greater. Any resistant species surviving at this time would need to be present in sufficient numbers within the host, to prevent dilution of resistant alleles by susceptible larvae ingested from pasture. Perhaps more importantly for IVM resistance development, routine use of oral moxidectin on this farm at lambing time may have influenced the IVM phenotype of the *T. circumcincta* population. The persistent effect of moxidectin prevents establishment by *H. contortus* and *T. circumcincta* larvae for up to five weeks post-treatment (Kerboeuf et al., 1995), but it does not target immature larvae of other species (Zoetis product datasheet, Cydectin ^®^ 0.1% w/v oral solution), decreasing the macrocyclic lactone selection pressure on the wider strongyle community.

The FECRT was followed up using two additional tests of phenotypic resistance: an EHT for BZ resistance and a LDT for IVM. The EHT has been studied extensively using laboratory isolates of various species, and has also been utilised in some field studies (Calvete et al., 2014). An EHT differentiates between resistant and susceptible populations by the known ability of TBZ to prevent hatching of BZ susceptible eggs (Le Jambre, 1976). The EHT results reported in this study were consistent with the FECRT data. By comparing the ED_50_ of the pre- and post-BZ treatment assays, a considerable increase was noted in the concentration at which 50% of the eggs failed to hatch, from 0.048 μg/ml to 0.615 μg/ml TBZ. Although a full EHT was not carried out post-IVM, a three-fold increase was observed in the percentage hatch in the wells containing 0.1 μg/ml TBZ, following *in vivo* IVM treatment, suggesting a dual-resistant population. Following ring testing of the EHT, von Samson-Himmelstjerna et al (2009) suggested that the presence of a resistant sub-population might be detected by recording the percentage hatch in the 0.2 and 0.3 μg/ml wells. Interestingly, in our pre-treatment EHTs we noted 31% and 24% egg hatch at these concentrations respectively, similar percentages to the proportion of *T. circumcincta* represented.

In contrast, the LDT was less informative, possibly related to the small number of replicates used in the post-IVM test. Although each of the pre-FECRT LDTs were comparable with each other, suggesting the LDT was repeatable, the fold-change post-IVM treatment was small (RR: 1.7). Two controls were used in the LDT; 2% DMSO and deionised water. Despite hatching of almost all eggs, development to L3 in the 2% DMSO control wells could be as low as 53%, with higher levels of development recorded in the water wells. However, these results are similar to others reported in the literature: rates of larval development varied from 64 to 86% for the L3 of *Oesophagostomum* species in 6% DMSO control wells (Varady et al., 1996) and from 58 to 89% for *H. contortus,* in control wells containing no DMSO (Heim et al., 2015). The use of a micro agar LDT, rather than a liquid based assay, is reported to support higher levels of development, ranging from 85 to 95% (Demeler et al., 2013; Gill et al., 1995). However, in the present study, up to 86% of eggs were capable of developing to L3 post-IVM treatment in the 2% DMSO control wells, suggesting variation in rates of development also reflects the mixed species present (Calvete et al., 2014). A similar difference was also noted in the 0.5% DMSO control wells of the EHT, with a lower hatch rate pre-BZ treatment compared with post-BZ treatment.

Pyrosequencing of the three BZ resistance associated codons of the β-tubulin isotype-1 gene (200, 198 and 167), found no significant differences between sampling time points in the BZ or IVM treated *T. circumcincta* populations. The numbers of individuals genotyped at each sampling time point was small compared with the effective population size, and this may explain the appearance of genotypes on days 7 and 14 which were not noted on day 0, including individuals with resistance associated polymorphisms at codon 198.

The pre-BZ treatment sample had a high frequency (∼73%) of resistance-associated mutations. BZ resistance is thought to be a recessive trait in *H. contortus* (Kwa et al., 1995), and in a previous study, no *H. contortus* adults that were heterozygous for the resistance mutation at codon 200 (with susceptible genotypes at codons 198 and 167) were found post-albendazole treatment (Barrère et al., 2012). In this study, we genoyped the offspring of *T. circumcincta* adult survivors, and in contrast to Kwa et al (1995), detected the presence of individuals with fully susceptible genotypes post-BZ treatment. This suggests that *T. circumcincta* adults that are heterozygous for resistance mutations have survived to produce offspring with susceptible genotypes. Consistent with our findings, a study by Keegan et al. (2017), in Ireland, found *T. circumcincta* adults surviving oxfendazole treatment which were heterozygous at codon 200 alone (P200 F/Y, P198E, P167 F). Interestingly, they also identified individuals with fully susceptible genotypes, consistent with the presence of an additional BZ resistance locus, which could explain the differences in heterozygote survival described above.

Post-BZ treatment the increase in individuals with resistance associated polymorphisms at codon 198, indicates that these genotypes were selected by BZ treatment in addition to resistance associated mutations at codons 200 and 167. Interestingly, a combined EHT/LDT using *H. contortus*, suggested that, in larval stages at least, individuals with resistance associated polymorphisms at codon 198 (P198 A) showed a higher level of resistance to TBZ than those with polymorphisms at codon 200 (P200Y) (Kotze et al., 2012). It is thought that individuals cannot carry more than one BZ-resistance associated mutation on the same haplotype at either position 200, 198 or 167 as this may be a lethal genotype (Barrère et al., 2012). While pyrosequencing does not provide any haplotypic data, analysis of 180 individuals in this project did not detect any larvae carrying more than two BZ resistance-associated SNPs, suggestive of no more than one resistant codon per allele. Post-IVM treatment, offspring of surviving adults share the same genotypes as those sequenced following BZ treatment, including those which are homozygous resistant at codon 200 indicating the presence of a dual-IVM and BZ resistant *T. circumcincta* population on the farm.

Guidelines are available in the UK to assist farmers in reducing worm burden and controlling PGE in their flocks (Abbott et al., 2012). These are continually reviewed and updated based on current evidence with a view to increasing production efficiency, improving welfare, and reducing the rate of development of resistance (Learmount et al., 2015). Currently, molecular markers are available for BZ resistance (Kwa et al., 1995; Silvestre and Cabaret, 2002; Ghisi et al., 2007; Redman et al., 2015), and markers are being further characterised for levamisole resistance (Boulin et al., 2011; Barrère et al., 2014). It is possible to use the BZ resistance genetic markers to assess the presence and origin of resistance in field populations (Redman et al., 2015), but little is known about IVM resistance, which is thought to be polygenic (Kotze et al., 2014). Modern methods of parasite speciation such as the nemabiome (Avramenko et al., 2017) and multiplex tandem PCR (Roeber et al., 2017) offer promise for the future diagnosis of anthelmintic resistant worms. However, until the use of such molecular tools becomes widespread, FECRT will continue to provide a useful measure of drug efficacy, but in the absence of species prevalence data, must be interpreted with care. The presence of a dual-resistant *T. circumcincta* population on this farm indicates that the use of either anthelmintic will preferentially select for this pathogenic species, and that combination treatments of BZ and IVM, or rotation of anthelmintic classes may not be beneficial for this flock.

## 5 Conclusion

This study clearly demonstrates the complicating factors of strongyle species diversity in field populations when interpreting a FECRT. We would therefore encourage the routine speciation of strongyles obtained during both pre- and post-treatment FEC samples, for optimal interpretation of the results, with the additional testing of a combination of products for each group of lambs used in the FECRT to enable detection of dual-resistant species. Pyrosequencing gave additional information but would be unlikely to provide significant insight to a farmer in practice except in the early stages of BZ resistance (*e.g.* for *N. battus* (Morrison et al., 2014)); however, pyrosequencing would be useful for monitoring management strategies in the research setting, or if a BZ drench was not used during the FECRT.
