# Supplementary material for: Hidden in plain sight - Multiple resistant species within a strongyle community
Source: Vet Parasitol. 2018 Jul 15;258:79–87. doi: 10.1016/j.vetpar.2018.06.012 (PMC6052248; doi:10.1016/j.vetpar.2018.06.012)
Supplement: Supplementary file 1 [file mmc1.docx]

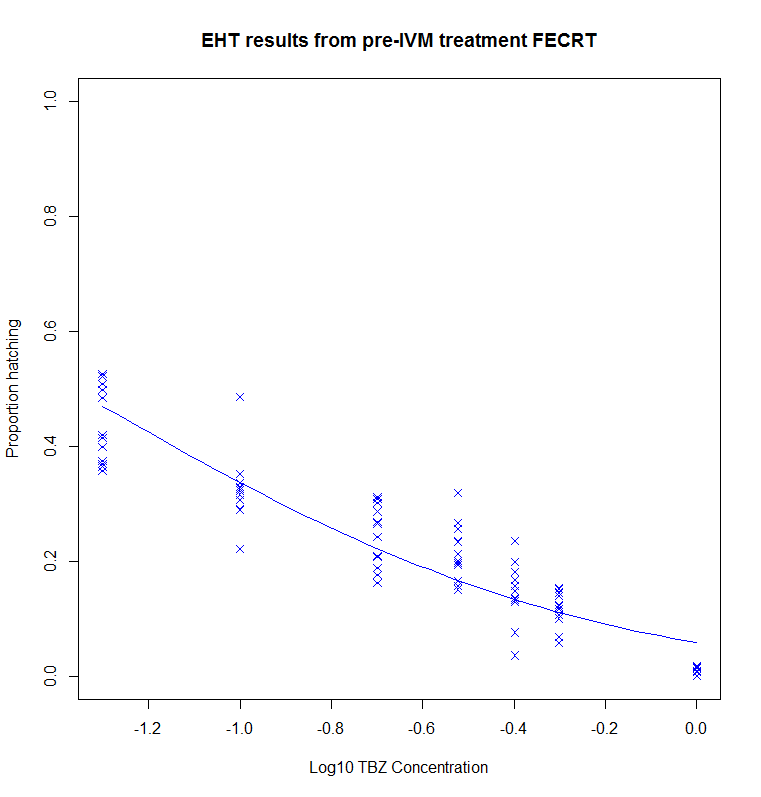
**Supplementary figure 1: Egg hatch test: pre-ivermectin faecal egg count reduction test** The corrected EHT data is shown, with log_10_(thiabendazole concentration (µg/ml)) plotted against the proportion hatching. The data was modelled in R using a binomial GLM (probit), and the regression line is plotted here. Only two 0.1 µg/ml wells were included post-ivermectin treatment and so the post-ivermectin data is not included here.


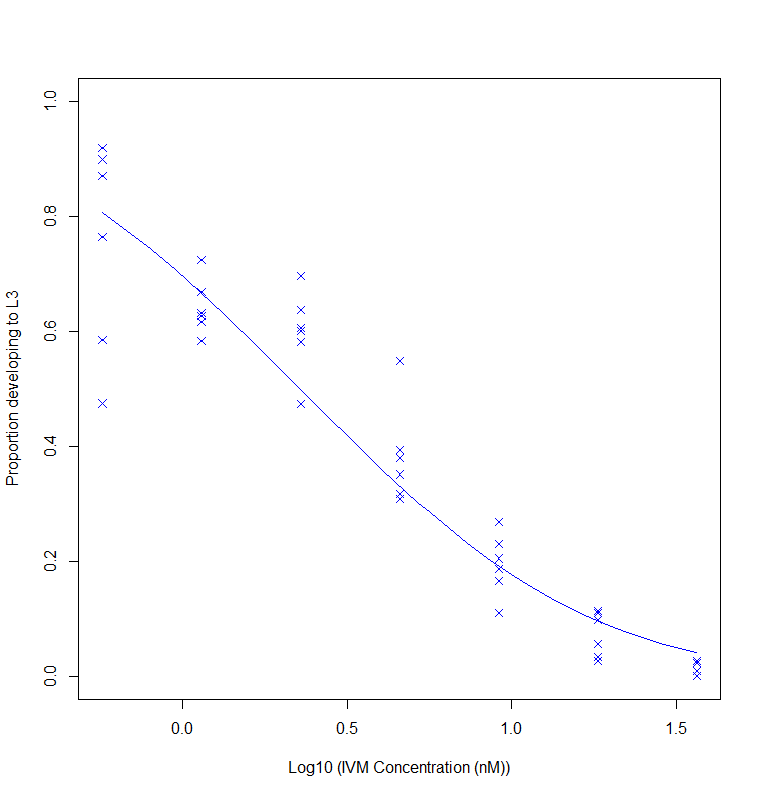
**Supplementary figure 2: Larval development test: pre-benzimidazole faecal egg count reduction test** The corrected LDT data is shown, with log_10_(ivermectin concentration (nM)) plotted against the proportion developing to L3. The data was modelled in R using a binomial GLM (probit), and the regression line is plotted here. No LDT was carried out post-benzimidazole treatment due to a lack of eggs.
