# Supplementary material for: Hidden in plain sight - Multiple resistant species within a strongyle community
Source: Vet Parasitol. 2018 Jul 15;258:79–87. doi: 10.1016/j.vetpar.2018.06.012 (PMC6052248; doi:10.1016/j.vetpar.2018.06.012)
Supplement: Supplementary file 2 [file mmc2.docx]

| Assay plate | Average percentage hatch |
| --- | --- |
|  | **0.5% DMSO** |
| Post-BZ FECRT | 91 |
| Pre-BZ FECRT  (4 plates) | 79 |
| Pre-IVM FECRT  (4 plates) | 85 |

**Supplementary Table 1: Control wells of the egg hatch tests.** The average percentage hatch in the 0.5% DMSO control wells is reported for each sample population tested.

| Assay plate | Average percentage development to L3 | | Average percentage hatch | |
| --- | --- | --- | --- | --- |
|  | **Water** | **2% DMSO** | **Water** | **2% DMSO** |
| Post-IVM FECRT | 92 | 86 | 95 | 92 |
| Pre-IVM FECRT, plate 1 | 78 | 58 | 99 | 98 |
| Pre-IVM FECRT, plate 2 | 66 | 53 | 96 | 96 |
| Pre-BZ FECRT, plate 1 | 87 | 75 | 98 | 97 |
| Pre-BZ FECRT, plate 2 | 85 | 67 | 98 | 97 |

**Supplementary Table 2: Control wells of the ivermectin larval development tests.** The average percentage hatch and development to L3 are shown for comparison between the water and 2% DMSO control wells for the ivermectin larval development tests.
